# Synthesis and cloxacillin antimicrobial enhancement of 2-methylsulfonylimidazolyl-1,4-dihydropyridine derivatives

**Published:** 2010

**Authors:** T. Akbarzadeh, A. Fallah Tafti, N. Samadi, A. Foroumadi, M. Amanlou, M. A. Faramarzi, A. Shafiee

**Affiliations:** 1 Department of Medicinal Chemistry; 2 Department of Drug and Food Control; 3 Drug Design & Development Research Center; 4 Department of Pharmaceutical Biotechnology, Faculty of Pharmacy; 5 Pharmaceutical Sciences Research Center, Tehran University of Medical Sciences, Tehran, Iran

**Keywords:** Multidrug-resistant *Staphylococcus aureus*, Cloxacillin, dihydropyridines

## Abstract

**Background and the purpose of the study:**

Hospital-acquired methicillin-resistant *Staphylococcus aureus* (MRSA) has been a major problem worldwide in chemotherapy of infection disease. This study was designed to assess the enhancing effects of a new group of dihydropyridine-3,5dicarboxamides, in combination with cloxacillin with distinctly different mechanisms of action against MRSAs.

**Material and methods:**

Dihydropyridine-3,5-dicarboxamides with 2-methylsulfonylimidazole at 4 position 6a-k were synthesized by the reaction of corresponding aldehyde 5 with different N-aryl acetoacetamides 3 in the presence of ammonium hydroxide. Agar disc diffusion method was used to determine the antibacterial and potentiating activity of different synthetic compounds in the presence and absence of cloxacillin to evaluate their activity as modulators of multidrugresistant (MDR).

**Results and major conclusion:**

The antibacterial effect of cloxacillin was enhanced by compounds 6g and 6h against cloxacillin-resistant strains (MRSA_1_ and MRSA_2_). The potentiation was found 1 2 to be statistically significant (p<0.01). Compound 6g at concentration of 1000 µg/disc, caused a 329 percent potentiation of the activity of cloxacillin against MRSA_1_.

## INTRODUCTION

One of the major problems in hospitals and community is the emergence and spread of MDR organisms such as *Staphylococcus aureus* that display resistance to methicillin; cloxacillin and other narrow-spectrum beta-lactamase-resistant penicillin antibiotics as well as cephalosporins (1, 2). These bacteria are labeled as methicillin-resistant *Staphylococcus aureus* (MRSA). The hospital MRSA is usually multi-drug resistant, demonstrating resistant to other classes of antibiotics. Overuse of antibiotics and the use of improper drugs may be some of the reasons for development of virulent strains. Infections with these organisms represent a serious challenge to the practitioner as therapeutic options are limited and associated mortality is high (3). Many institutions have observed an increase in blood culture isolates of MRSA over the past two decades.

There is a clear and urgent need to discover and develop new effective and non-toxic drugs that are able to overcome MDR. Some of the medicines have been reported to enhance the antibacterial activity of different antibiotics against different resistant strains (4–6). 1,4-Dihydropyridine derivatives have been found as multidrug-resistance (MDR) reversal agents in tumor cells (7). In continuing investigation on 1,4-dihydropyridine compounds (8, 9) and to characterize new synthetic compounds with activity as modulators of MDR in *Staphylococcus aureus* (10), some new N, N-diaryl-2,6-dimethyl-4-(2methyl-sulfonylimidazol5-yl)-1,4-dihydropyridine 3,5-dicarboxamide derivatives were synthesized and their antibacterial properties were evaluated.

## MATERIAL AND METHODS

### 

#### Chemistry

Melting points were determined using a Kofler hot stage apparatus and are uncorrected. ^1^H-NMR spectra were run on a Bruker FT-80 and FT-500 spectrometers (Brukers, Rheinsteetteen, Germany). TMS was used as internal standard. Mass spectra were measured with a Finnigan TSQ-70 spectrometer (Finnigan Mat, Bremen, Germany). Infrared spectra were acquired on a Nicolet 550-FTIR spectrometer (Maidson, WI, USA).

**Scheme 1:**
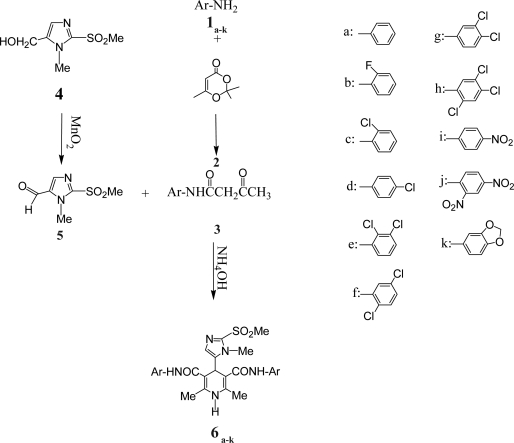


The synthesis of 1,4-dihydropyridine derivatives **6a-k** was achieved following the steps outlined in Scheme 1. Reaction of amine **1** with 2,2,6-trimethyl-4H-1,3-dioxine-4-one **2** afforded the corresponding acetoactamide **3** (76-92% yield) (11). Oxidation of **4** with manganese (IV) oxide in chloroform afforded the desired aldehyde **5** in 90% yield (12, 13). Symmetrical dihydropyridine analogues **6** were prepared by classical Hantzsch condensation in which the aldehyde **5** was reacted with the corresponding N-aryl actoacetamide **3a-k** and ammonium hydroxide in ethanol.

#### Antibacterial and enhancement effect of the synthesized compounds

Minimum inhibitory concentration of cloxacillin was determined by conventional agar dilution method against 2 clinically isolated cloxacillin-resistant *Staphylococcus aureus*.

For determination of enhancing effect of the synthetic compounds, a stock solution of cloxacillin was prepared in dimethylsulfoxide (DMSO; 1 ml) which was added to molten Mueller-Hinton (MH) agar (19 ml) at 50°C to give the sub-inhibitory concentration of 12.5 µg/ml. The bacteria inocula were prepared by suspending overnight colonies from MH agar media in 0.85% saline. The inocula were adjusted photometricaly at 600 nm to a cell density equivalent to approximately 0.5 McFarland standard (1.5×10^8^ CFU/ml) (10, 14). MH agar plates were seeded individually with bacterial suspensions using a sterile cotton swab. Two-fold dilution of the test compounds were prepared in DMSO and 20 µl of each dilute was added to blank discs to give the final concentrations of 1000, 500, 250 and 125 µg/disc. Discs containing synthetic compounds were applied on the surface of seeded MH agar plates. Similar loaded discs were applied to agar plates without any antibacterial agents. To insure that the solvent had no effect on bacterial growth, a control test was performed with test medium supplemented with DMSO at the same dilutions as used in the experiment. Blank discs containing 20µl DMSO were used as negative controls. The plates were incubated at 30-35°C for 18 hrs. After incubation, the mean inhibition zone diameter for each concentration was determined. The diameters of inhibition zones produced due to individual and combined effects of effective compounds were recorded. The increase in the surface area (Πr2) due to a combination of effects was statistically evaluated by determining student's t-test for its level of significance (15).

#### General procedure for the synthesis of N, N′-diaryl-2,6-dimethyl-4-(2-methylsulfonyl-1-methylimidazol 5-yl)-1,4-dihydropyridine-3,5-dicarboxamides (6_a-k_).

A solution of ammonium hydroxide (26%, 0.5 ml) was added to a stirring solution of compound **4** (240 mg, 1.26 mmoles) and corresponding N-arylacetoacetamide **3** (2.54 mmoles) in methanol (5 ml). The mixture was protected from light and refluxed under argon overnight. After cooling the precipitate was filtered and crystallized from methanol to give the title compounds **6**.

The physical data for compounds **6a-k** are given in Table 1.

**Table 1:** Physical data for compounds **6a-k**

Comp.	Ar	Yield (%)	m.p. (°C)	Physical data
**6a**	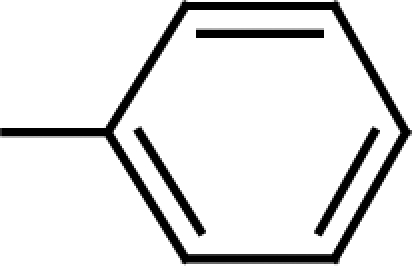	52	238–239	IR (KBr): 1648(NHC=O), ^1^HNMR (DMSO-d_6_) δ: 9.52 (s, 2H, NH), 8.22 (s, 1H, N_1_-H DHP), 7.56 (d, J=7.6 Hz, 4H, aromatic), 7.26 (t, J=7.6 Hz, 4H, aromatic), 7.02 (t, J=7.6 Hz, 2H, aromatic), 6.98 (s, 1H, C_4-_H imidazole), 5.15 (s, 1H, C_4-_H DHP), 3.84 (s, 3H,NCH3), 3.13 (s, 3H, SO2CH3), 2.09 (s, 6H, 2,6 CH_3_). Mass: m/z (%) 504 (M^+^-1, 0.5%), 385 (43), 371 (98), 292 (100), 253 (98), 186 (70), 160 (97), 119 (64).
**6b**	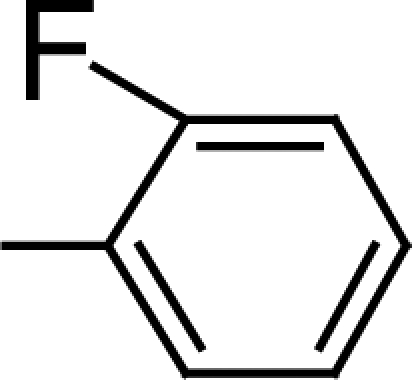	54	230–231	IR (KBr): 1649(NHC=O), ^1^HNMR (DMSO-d_6_) δ: 9.27 (s, 2H, N-H), 8.47 (s, 1H, N_1_-H DHP), 7.14 (m, 8H, aromatic), 6.84 (s, 1H, H-C_4_ imidazole), 5.14 (s, 1H,H-C_4_ DHP), 3.87 (s, 3H, N-CH_3_), 3.40 (s, 3H, SO_2_-CH_3_), 2.15 (s, 6H, 2,6 CH_3_). Mass: m/z (%) 541 (M^+^, 3), 463 (47), 325 (61), 292 (100), 214 (72), 188 (100), 137 (100), 80 (100).
**6c**	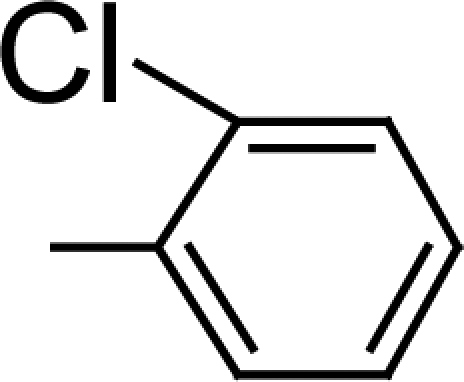	44	231–232	IR (KBr): 1654(NHC=O), ^1^HNMR (DMSO-d_6_) δ: 9.08 (s, 2H, N-H), 8.46 (s, 1H, N_1_-H DHP), 7.50 (dd, J=8Hz, J=1.5 Hz, 2H, aromatic), 7.45 (dd, J=8Hz, J=1.5 Hz, 2H, aromatic), 7.29 (td, J=8Hz, J=1.5 Hz, 2H, aromatic), 7.17 (td, J=8Hz, J=1.5 Hz, 2H, aromatic), 6.87 (s, 1H, C_4_-H imidazole), 5.16 (s, 1H, - C_4_-H DHP), 3.90 (s, 3H, N-CH_3_), 3.31 (s, 3H, SO_2_-CH_3_), 2.20 (s, 6H, 2,6 CH_3_), Mass: m/z (%) 574 (M^+^+1, 7), 495 (48), 448 (40), 420 (27), 341 (100), 266 (100), 186 (100), 106 (100).
**6d**	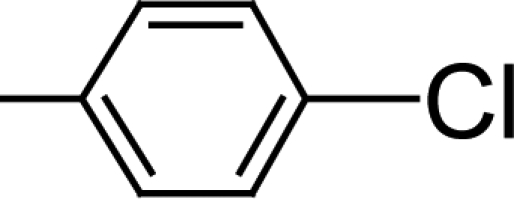	51	248–249	IR (KBr): 1629(NHC=O), ^1^HNMR (DMSO-d6) δ: 9.65 (s,2H, N-H), 8.44 (s, 1H, N_1_-H DHP), 7.60 (d, J=9 Hz, 4H, aromatic), 7.30 (d, J=9 Hz, 4H, aromatic), 6.82 (s, 1H, C_4_-H imidazole), 5.12 (s, 1H, C_4_-H DHP), 3.83 (s, 3H, N-CH_3_), 3.31 (s, 3H, SO2-CH_3_), 2.09 (s, 6H, 2,6 CH_3_). Mass: m/z (%) 573 (M^+^, 2), 495 (32), 342 (58), 295 (100), 226 (60), 188 (100), 98 (100), 55 (100).
**6e**	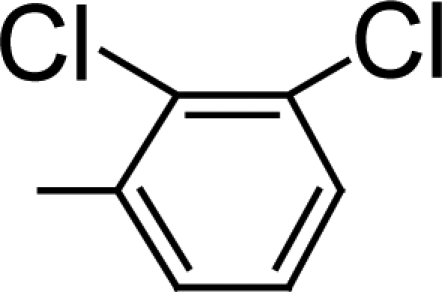	68	211–212	IR (KBr): 1619(NHC=O), ^1^HNMR (DMSO-d_6_) δ: 9.35 (bs, 2H, NH), 8.53 (s, 1H, N_1_-H DHP), 7.45 (m, 4H, aromatic), 7.31 (t, 2H, aromatic), 6.86 (s, 1H, C_4_-H imidazole), 5.17 (s, 1H, C_4_ -H DHP), 3.89 (s, 3H, NCH_3_), 3.34 (s, 3H, SO_2_CH_3_), 2.19 (s, 6H, 2,6 CH_3_). Mass: m/z (%) 644 (M^+^+3, 3), 641 (M^+^,0.5), 563 (28), 482 (60), 375 (100), 322 (85), 266(100), 160 (100).
**6f**	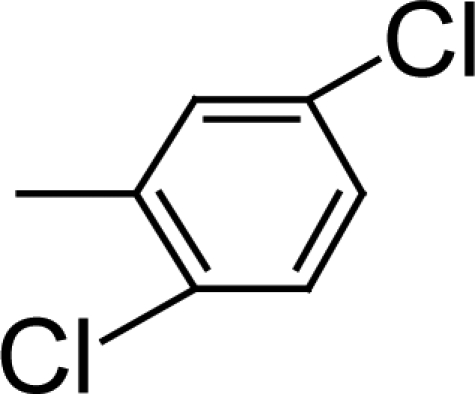	88	228–229	IR (KBr): 1622(NHC=O), ^1^HNMR (DMSO-d_6_) δ: 9.15 (s, 2H, NH), 8.62 (s, 1H, N_1_-H DHP), 7.69 (d, J=2.5 Hz, 2H, C_6_ -H phenyl), 7.50 (d, J=9 Hz, 2H, C_3_ -H phenyl), 7.26 (dd, J=9 Hz, J=2.5 Hz, 2H, C_4_-H phenyl), 6.87 (s, 1H, C_4_-H imidazole), 5.14 (s, 1H, C_4_-H DHP), 3.90 (s, 3H, NCH_3_), 3.33 (s, 3H, SO_2_CH_3_), 2.20 (s, 6H, 2,6 CH_3_). Mass: m/z (%) 644 (M^+^+3, 3), 641 (M^+^,1), 565 (49), 482 (58), 375 (100), 266 (100), 186(100), 109 (100).
**6g**	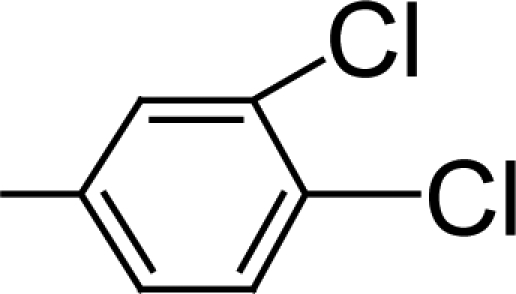	89	263–264	IR (KBr): 1624(NHC=O), ^1^HNMR (DMSO-d_6_) δ: 9.80 (s, 2H, NH), 8.59 (s, 1H, N_1_-H DHP), 7.94 (s, 2H, aromatic), 7.53 (s, 4H, aromatic), 6.81 (s, 1H, C_4_-H imidazole), 5.11 (s, 1H, C_4_-H DHP), 3.812 (s, 3H, NCH_3_), 3.33 (s, 3H, SO_2_CH_3_), 2.10 (s, 6H, 2,6 CH3). Mass: m/z (%) 644 (M^+^+3, 2), 641 (M^+^,0.5), 375 (28), 321 (45), 266 (67), 188 (100), 159(100), 106 (100).
**6h**	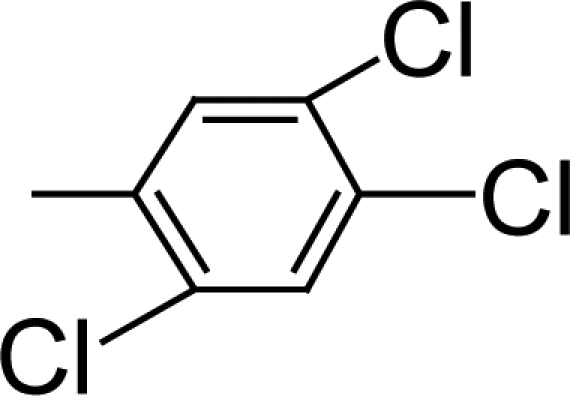	98	152–153	IR (KBr): 1670(NHC=O), ^1^HNMR (DMSO-d_6_) δ: 9.23 (s, 2H, NH), 8.65 (s, 1H, N_1_-H DHP), 7.89 (s, 2H, aromatic), 7.87 (s,2H, aromatic), 6.86 (s, 1H, C_4_-H imidazole), 5.12 (s, 1H, C_4_-H DHP), 3.89 (s, 3H, NCH_3_), 3.34 (s, 3H, SO_2_CH_3_), 2.20 (s, 6H, 2,6 CH_3_). Mass: m/z (%) 709 (M_+_, 7), 516 (2), 410 (24), 295 (20), 223 (64), 198 (32), 153(100), 109 (100).
**6i**	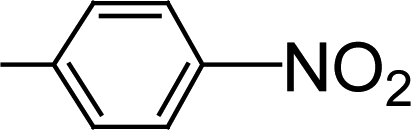	67	163–164	IR (KBr): 1680(NHC=O), ^1^HNMR (DMSO-d_6_) δ: 10.14 (s, 2H, NH), 8.74 (s, 1H, N_1_-H DHP), 8.18 (d, J=9 Hz, 4H, aromatic), 7.83 (d, J=9 Hz, 4H, aromatic), 6.84 (s, 1H, C_4_-H imidazole), 5.17 (s, 1H, C4-H DHP), 3.84 (s, 3H, NCH_3_), 3.32 (s, 3H, SO_2_CH_3_), 2.14 (s, 6H, 2,6 CH3). Mass: m/z (%) 595 (M^+^, 2), 564 (9), 456 (40), 318 (17), 292 (100), 266 (30), 212(33), 108 (68).
**6j**	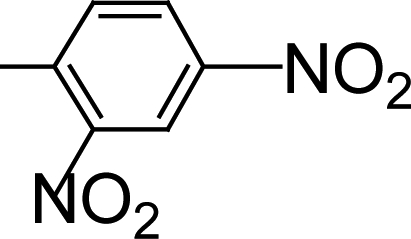	97	200–201	IR (KBr): 1680(NHC=O), _1_HNMR (DMSO-d_6_) δ: 8.64 (d, J=2.5 Hz, 2H, C3-H phenyl), 8.45 (dd, J=9 Hz, J=2.5 Hz, 2H, C5-H phenyl), 7.89 (d, J=9 Hz, 2H, C6-H phenyl), 6.84 (s, 1H, C4-H imidazole), 5.11 (s, 1H, C4-H DHP), 3.81 (s, 3H, NCH_3_), 3.29 (s, 3H, SO_2_CH_3_), 2.27 (s, 6H, 2,6 CH3). Mass: m/z (%) 685 (M^+^, 2), 502 (37, 475 (12), 411 (10), 318 (15), 293 (100), 183 (27), 81 (40).
**6k**	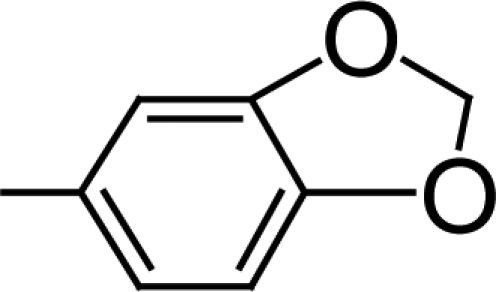	41	200–202	IR (KBr): 1623(NHC=O), ^1^HNMR (DMSO-d_6_) δ:9.42 (s, 2H, NH), 8.14 (s, 1H, N_1_-H DHP), 7.24 (d, J=2 Hz, 2H, aromatic), 6.95 (dd, J=8.3 Hz, J=2 Hz 2H, aromatic), 6.82 (s, 1H, C4-H imidazole), 6.80 (d, J=8.3 Hz, 2H, aromatic), 5.95 (s, 2H, O-CH_2_-O), 5.09 (s, 1H, C4-H DHP), 3.82 (s, 3H, NCH_3_), 3.37 (s, 3H, SO_2_CH_3_), 2.07 (s, 6H, 2,6 CH3). Mass: m/z (%) 685 (M^+^-1, 18), 434 (21), 394 (28), 308 (100), 258 (63), 180 (100), 109 (100), 50 (100).

## RESULT AND DISCUSSION

The MIC value of 50 µg/ml for cloxacillin was obtained by agar dilution method for two clinically isolated cloxacillin-resistant organisms (MRSA_1_, MRSA_2_). A sub-inhibitory concentration of cloxacillin (12.5 µg/ml) was selected for further experiments. The antibacterial activity of synthetic compounds was evaluated against MRSA_1_ and MRSA_2_. No inhibitory effect was observed at all concentrations ≤1000 µg/disc. Synthesized compounds were evaluated for bacterial resistance modifying activity of cloxacillin against two MRSA strains using the agar disc diffusion method.

Compound **6g** showed enhancing effect with sub-inhibitory concentration of cloxacillin (12.5 µg/ml) at all tested concentrations and compound **6h** enhanced antimicrobial activity of cloxacillin at concentration of 250 µg/disc or higher. None of the other synthetic compounds showed any effect. As cloxacillin was used in a sub-inhibitory concentration (12.5 µg/ml) and compounds **6g** and **6h** did not show any antibacterial activity in the absence of cloxacillin, the appearance of inhibition zones in combination of cloxacillin and synthetic compounds indicated the enhancing effect of the compounds and it was found to be statistically significant (p<0.01).

**Table 2:** Increase (%) in effects of cloxacillin with effective synthetic compounds compared with their individual activity based on the surface area of inhibition zone.

Compound No.	Concentration µg/disc	Bacteria	Mean diameter of the inhibition zone (mm) colcoxacillin (12.5 µg/disc)+synthetic compounds	Percentage increase on the basis of xr^2(a)^
**6g**	1000	MRSA_1_	14.5	329
	500	MRSA_1_	12	194
	250	MRSA_1_	10	104
	125	MRSA_1_	10	104
	1000	MRSA_2_	13	244
	500	MRSA_2_	12	194
	250	MRSA_2_	9	65
	125	MRSA_2_	9	65
**6h**	1000	MRSA_1_	11	147
	500	MRSA_1_	9	65
	250	MRSA_1_	9	65
	125	MRSA_1_	-	-
	1000	MRSA_2_	11	147
	500	MRSA_2_	10	104
	250	MRSA_2_	-	-
	125	MRSA_2_	-	-

(a) Mean surface area of the inhibition zone (mm^2^) was calculated as Πr^2^ on the basis of the mean diameter (2r) and% increase was calculated as (B^2^-A^2^)/A^2^×100, where A is surface area due to individual synthetic compound effect, (A=7, the individual synthetic compound was inactive), B=surface area due to combined effect; These were calculated statistically by determining ‘t’ Test two sample assuming unequal which showed the differences to be highly significant (P<0.01).

Compound **6g** significantly restored the bacterial sensitivity to the antibiotic which was showed 330 percent increase in the inhibition zone area on the cloxacillin supplemented plates when applied in 1000 µg/ml. However in the lower concentration of **6g** (500 µg/ml) against MRSA_1_ and at 1000 µg/ml and 500 µg/ml against MRSA_2_ the inhibition zones showed 190-250 percent potentiation increase. Compound **6h** showed enhancing effect at concentrations of 250 µg/ml or higher (60-150 percent potentiation). The results demonstrated that modification of classical structure of 3,5-diester of 1,4-dihydropyridines to 3,5-diamides could be a good way to combat cloxacillin resistant strains. Comparison of the activities of symmetrical amides indicates that the presence of electron withdrawing groups on the phenyl ring is essential for enhancement of the activity of cloxacillin. It seems that presence of halogen at 3 and 4 positions of phenyl in 3,5-diarylcarboxamide structure resulted in enhancement of the activity. However existence of a substitute on ortho position of phenyl reduced the effect. It is noteworthy to mention that the cause of enhancing effect is not known and require further investigation. Though several inverstigations have been carried out to overcome the microbial resistance by combination of synthetic and natural compounds with different antibiotics against resistant bacterial strains (4–6, 10), this paper is the first report on the potentiating effect of cloxacillin antibacterial activity with 1,4-dihydropyridine derivatives. It is also of interest to continue this study by modifying other parts of the structure in order to find more potent modulating agents.
